# Intestinal Carriage of Extended-Spectrum *β*-Lactamase- (ESBL-) Possessing *Escherichia coli* and *Klebsiella* Species among Nepalese Health Science and Non-Health Science Students

**DOI:** 10.1155/2021/4767429

**Published:** 2021-04-09

**Authors:** Bhawana Sapkota, Santosh K. Yadav, Gunaraj Dhungana, Shamshul Ansari, Shyam K. Mishra

**Affiliations:** ^1^Department of Medical Laboratory Technology, JF Institute of Health Sciences, Kathmandu, Nepal; ^2^Department of Microbiology, Rajarshi Janak University, Janakpurdham, Nepal; ^3^Department of Microbiology, Chitwan Medical College, School of Medicine, Bharatpur, Nepal; ^4^Department of Microbiology, Maharajgunj Medical Campus, Institute of Medicine, Tribhuvan University, Kathmandu, Nepal; ^5^School of Optometry and Vision Science, University of New South Wales Sydney, Kensington, Australia

## Abstract

Infections due to extended-spectrum* β*-lactamase- (ESBL-) producing Gram-negative bacteria have led to increased mortality, morbidity, and economic burden worldwide. These bacteria can colonize the healthy intestine of human beings and can disseminate in communities and hospital. This study aimed to investigate the prevalence of fecal carriage of ESBL-producing *Escherichia coli* and *Klebsiella* species among health science (HS) and non-health science (NHS) students. This descriptive cross-sectional study was conducted on 104 HS and 104 NHS students in which one stool sample from each student was collected and processed for bacterial culture and sensitivity testing according to standard bacteriological procedures. Each morphotype was identified and characterized phenotypically. The antimicrobial sensitivity profile of bacterial isolates was determined by the Kirby–Bauer disk diffusion technique. ESBL production was tested by combination disk method as recommended by the Clinical and Laboratory Standards Institute. Out of 208 stool samples, *E. coli* and *Klebsiella* spp. were recovered from 203 (86.8%) and 31 (13.2%) stool samples, respectively. Among those 234 isolates, 69 were positive for ESBL which included *E. coli* (*n* = 66, 95.7%) and *Klebsiella* spp. (*n* = 3, 4.3%). Fifty (42.4%) out of 118 isolates from HS students and 19 (16.4%) out of 116 from NHS students were colonized by ESBL-producers. Compared to non-ESBL producers, a higher number of ESBL-producing isolates were resistant to ciprofloxacin (14.5% vs. 1.8%, *p* < 0.001), cotrimoxazole (59.4% vs. 16.4%, *p* < 0.001), and amikacin (10.1% vs 4.2%, *p* < 0.001). All *E. coli* and *Klebsiella* species isolates were susceptible to meropenem. The prevalence of fecal carriage of ESBL-producing bacteria was higher in HS students; however, there was a considerable number of these strains colonizing NHS students as well. This “iceberg phenomenon” of asymptomatic carriage of ESBL-producing pathogens might act as a source of infection in both the community and hospitals. Therefore, surveillance of carriage of drug-resistant bacteria should be performed regularly.

## 1. Introduction

The gut microbiota is an extremely complex community composed of both aerobic and anaerobic bacterial populations. However, in healthy humans, the gut microbiota is relatively stable and ingested microorganisms are cleared easily due to the presence of commensal microbiota [1]. Antibiotic resistance in *Escherichia coli* (*E. coli*) and *Klebsiella* species (common intestinal members of the microbiota) to third-generation cephalosporins are a major threat in hospitalized patients as well as in community populations. This resistance is mediated mainly by acquired extended-spectrum *β*-lactamase (ESBL) enzymes, which have the potential to hydrolyze penicillins, cephalosporins, and monobactams [2]. Rates of intestinal colonization by ESBL-producing *E. coli* and *Klebsiella* species have increased dramatically worldwide. This increased prevalence has been associated with the dissemination of specific clones and plasmids harboring ESBL genes. The spread of ESBL-producing organisms to healthy community residents is one of the most threatening epidemiological problems worldwide [3]. The rapid emergence of ESBL-producing bacteria results in a therapeutic burden that generates economic and public health concerns to healthcare systems globally. Thus, it is necessary to identify high-risk populations to help reduce this problem [4].

Although some data are available on the intestinal carriage of ESBL-producing Enterobacterales in community populations in South Asian countries [5–8], there is a lack of published estimates of intestinal carriage of ESBL-producing *E. coli* and *Klebsiella* species among health science (HS) and non-health science (NHS) students. Therefore, it was hypothesized that the HS students who are concerned with hospital patients and community populations would harbor greater numbers of antibiotic-resistant ESBL-producing *E. coli* and *Klebsiella* species as part of their commensal intestinal microbiota. They may also disseminate these bacterial populations causing hospital-associated as well as community-acquired infections. This study focused on the intestinal (fecal) carriage rate of ESBL-producing *E. coli* and *Klebsiella* species among HS and NHS students of a health science institute of central Nepal which provides undergraduate courses in nursing, pharmacy, and medical laboratory technology.

## 2. Materials and Methods

### 2.1. Study Design and Study Population

This descriptive cross-sectional study was conducted over six months among the HS and NHS students of the JF Institute of Health Sciences, Nepal, and microbiological procedures were carried out at the microbiology department of the JF Institute of Health Sciences/Little Angels College of Higher Studies (LACHS). From July to December 2017, a total of 208 students (104 each from HS and NHS categories) were enrolled. The HS students who participated in this study were from BNS (Bachelor of Nursing Science), B.Sc. Nursing (Bachelor of Science in Nursing), and B.Sc. MLT (Bachelor of Science in Medical Laboratory Technology) programs. Both BNS and B.Sc. Nursing fall under the nursing program, but they were recognized as different health science student groups in this study because only those people who have completed the certificate-level course in nursing can apply for the BNS, whereas for B.Sc. Nursing, candidates who have completed a higher secondary level in science can apply. All groups of HS students had frequent clinical posting in hospitals before the sampling period. Student volunteers not from the health science program of LACHS and who were interested to participate in the study were selected as the NHS students. The demographics of the participating students including previous antibiotic exposure or infections were recorded.

### 2.2. Inclusion and Exclusion Criteria

Students without prior antibiotic exposure for at least three months before study enrollment and those without infectious diseases were included in this study. The exclusion criteria included students who were under antibiotic therapy.

### 2.3. Stool Sample Collection and Processing

During the study, a total of 208 non-duplicate fecal samples were collected comprising of 104 each from the HS and NHS groups. After obtaining the written consent in local and English languages, each participant was provided with a clean, sterile, leak-proof, screw-capped plastic container with a plastic spoon and was instructed in the collection of a fresh stool sample. A single stool sample was collected from each student. Then, the fecal samples were processed for isolation and identification of *E. coli* and *Klebsiella* species according to standard microbiological methods recommended by the American Society for Microbiology (ASM) [9]. For this, each stool sample was inoculated onto a MacConkey agar plate and incubated aerobically at 37°C for 24 hours.

### 2.4. Identification of Bacterial Isolates

After incubation, different bacterial morphotypes were selected and further processed for identification by observation of colony characteristics, Gram's staining, motility, and different biochemical tests (catalase test, oxidase test, triple sugar iron agar test, indole production, citrate utilization, and urea hydrolysis) [9]. Those isolates identified as *E. coli* or *Klebsiella* species were subjected to antibiotic sensitivity testing and detection of ESBL production.

### 2.5. Antibiotic Sensitivity Testing

Antibiotic sensitivity testing (AST) was performed by the Kirby–Bauer disk diffusion method, and results were interpreted according to Clinical and Laboratory Standards Institute (CLSI) guidelines [10]. A loop was charged with at least 3–5 well-isolated colonies of bacteria, and these bacteria were transferred to a tube containing 5 ml of peptone water. The peptone water tube was incubated at 37°C until its turbidity matched a 0.5 McFarland standard. A sterile cotton swab was dipped into that peptone water, and the swab was rotated several times and then pressed firmly on the inner sidewall of the tube to remove excess inoculum from the swab. Then, the entire surface of a Mueller-Hinton agar (MHA) (HiMedia, India) plate was inoculated with the swab (by rotating the plate by 60° between streaking) to produce a lawn culture. The plates were left to dry for 10 minutes at room temperature with the lid closed and then antibiotic disks (ampicillin (10 *μ*g), piperacillin-tazobactam (100/10 *μ*g), cefotaxime (30 *μ*g), ceftazidime (30 *μ*g), meropenem (10 *μ*g), ciprofloxacin (5 *μ*g), cotrimoxazole (25 *μ*g), and amikacin (30 *μ*g)) (HiMedia, India) were placed on the surface of the agar plate and incubated in ambient air for 16–18 hours at 37°C. After incubation, the diameter of the zone of inhibition (ZOI) around each disk was measured and the isolate was classified as sensitive, of intermediate sensitivity, or resistant to each antibiotic using the CLSI zone size interpretation chart. *Escherichia* coli ATCC 25922 was used as the quality control strain for the test [10].

### 2.6. Detection of Extended-Spectrum-*β*-Lactamase- (ESBL-) Producing Isolates

Extended-spectrum-*β*-lactamase production in *E. coli* and *Klebsiella* species was detected following CLSI guidelines [10].

 
*Screening Test.* For ESBL screening, bacterial isolates were examined for their susceptibility to third-generation cephalosporins using ceftazidime (30 *μ*g) and cefotaxime (30 *μ*g) disks. If the ZOI was ≤22 mm for ceftazidime and/or ≤27 mm for cefotaxime, the isolate was considered a potential ESBL-producer [10].
 
*Confirmatory Test.* Confirmation of ESBL production was carried out using the combination disk test (CDT). In this test, ceftazidime (30 *μ*g) disk alone and in combination with clavulanic acid (30/10 *μ*g) or cefotaxime (30 *μ*g) disk alone and in combination with clavulanic acid (30/10 *μ*g) were applied onto a plate of MHA previously inoculated with the test strain. The plate was incubated in ambient air for 16–18 hours at 37°C. Isolates showing an increase of ≥5 mm in the zone of inhibition (ZOI) for either antimicrobial agent tested in combination with clavulanic acid versus the zone diameter of the agent when tested alone were considered positive for ESBL production. For standardization of the ESBL detection test, *K. pneumoniae* ATCC 700603 and *E. coli* ATCC 25922 were used as positive and negative controls, respectively [10].

### 2.7. Statistical Analysis

Data analysis was carried out using the SPSS version 16.0 and interpreted according to frequency distribution and percentage. Pearson's chi-square test was used to determine correlations between variables. *p* values ≤0.05 (95% CI) were considered to be statistically significant.

### 2.8. Ethical Statement

Ethical approval was obtained from the Ethical Review Board of Nepal Health Research Council, Kathmandu, Nepal (Reg. No. 254/2017). Informed consent was obtained from each student before enrollment in the study.

## 3. Results

### 3.1. Distribution of Enrolled Students and Bacterial Isolates

The total number of students who participated in the study was 208 which was equally split between the HS and NHS students (104 in each). Among all the students, 77 were male and 131 were female, giving a male to female ratio of 0.59. The mean age of the study participants was 21.37 years (SD ± 1.62) ranging from 18 years to 26 years.

A total of 234 isolates were grown. Of these, 86.8% were identified as *E. coli* (*n* = 203), 8.5% as *Klebsiella pneumoniae* (*n* = 20), and 4.7% as *Klebsiella oxytoca* (*n* = 11). One hundred and one (49.8%) and 102 (50.2%) isolates of *E. coli* were recovered from HS and NHS students, respectively. The recovery rate of *Klebsiella* spp. was 54.8% (*n* = 17) and 45.2% (*n* = 14) from HS and NHS students, respectively (Table 1). Among these students, 60, 25, and 19 were from B.Sc. Nursing, BNS, and B.Sc. MLT programs, respectively. Out of 101 *E. coli* isolated from HS students, 56.4% (*n* = 57), 24.8% (*n* = 25), and 18.8% (*n* = 19) were recovered from B.Sc. Nursing, BNS, and B.Sc. MLT students, respectively. Similarly, out of 17 *Klebsiella* species from HS students, 64.8% (*n* = 11) was recovered from B.Sc. Nursing and 17.6% (*n* = 3) from each BNS and B.Sc. MLT students (Table 2).

### 3.2. ESBL Carriage among Health Science and Non-Health Science Students

The overall prevalence of fecal carriage of ESBL-producing isolates among the students was 29.5%. Most (50 out of 118; 42.4%) isolates were from HS students and 19 out of 116 (16.4%) isolates were from NHS students. The rate of ESBL production among *E. coli* and *Klebsiella* species was 32.5% and 9.7%, respectively. ESBL production in *E. coli* was highest among HS students (46.5%, *p* < 0.001). Only a few isolates of *Klebsiella* species from HS students were ESBL-producers (*n* = 3, 17.6%). Among the NHS students, 18.6% (*n* = 19) *E. coli* were ESBL-producers, whereas none of the isolates of *Klebsiella* species produced ESBL (Table 3). Among the HS students, 45.6%, 56.0%, and 36.8% *E. coli* were ESBL positive from B.Sc. Nursing, BNS, and B.Sc. MLT students, respectively. Similarly, 33.3% (one out of three) and 18.2% (two out of 11) *Klebsiella* spp. isolates from BNS and B.Sc. Nursing students, respectively, were ESBL-producers (Table 4). None of the students harbored both ESBL-producing *E. coli* and *Klebsiella* spp.

### 3.3. Antibiotic Sensitivity Profiles of Bacterial Isolates

Both *E. coli* and *Klebsiella* spp. isolates from HS students had higher resistance rates than those from NHS students. Isolates of *E. coli* from HS students had increased resistance to ampicillin (71.3% vs 51.0%, *p*=0.001), third-generation cephalosporins (60.4% vs 30.4%, *p* < 0.001), and cotrimoxazole (39.6% vs 30.4%, *p*=0.005). Similarly, *Klebsiella* species from HS students had higher resistance rates to cotrimoxazole (29.4%) or to third-generation cephalosporins (23.5%) as all of the *Klebsiella* species isolates from NHS students were sensitive to all antibiotics except cotrimoxazole. All the isolates of *E. coli* and *Klebsiella* species from both HS and NHS students were susceptible to meropenem (Table 5). A higher number of ESBL-producers were resistant to ciprofloxacin (14.5% vs. 1.8%, *p* < 0.001), cotrimoxazole (59.4% vs. 16.4%, *p* < 0.001), and amikacin (10.1% vs 4.2%, *p* < 0.001) (Table 6).

## 4. Discussion

Antimicrobial resistance is a major public health issue which is likely to worsen in the coming decades. Gram-negative bacteria, particularly *E. coli* and *Klebsiella* species, are now often resistant to third-generation cephalosporins, and one of the reasons behind this is their capacity to produce ESBLs [1]. People colonized with ESBL-producing Gram-negative bacteria may transmit the resistant strains to other individuals, including hospitalized patients. High rates of intestinal carriage of such resistant bacteria have been described from developing countries [11, 12]. Various studies conducted worldwide have shown a dramatically elevated prevalence of ESBL-producing *E. coli* and *Klebsiella* species in fecal samples of healthy individuals [3, 13]. This study determined the fecal carriage rate of ESBL-producing *E. coli* and *Klebsiella* species among different clusters of students at the JF Institute of Health Sciences/Little Angels College of Higher Students in Nepal.

In this study, the overall fecal carriage rate of ESBL-producing *E. coli* and *Klebsiella* species among the enrolled students was 29.5% which is similar to previous reports of 25–38% ESBL carriage rate in healthy populations in Nepal [6] and Chad [14]. However, the result is much higher than some other reports from different parts of the world, for instance, 6.7% of student volunteers from Cameroon [12], 6.4% of healthy adults from Japan [15], 2.6% of medical students in Hungary [16], and 3.5% of healthy children in the USA [17]. A similar study conducted in Nepal in 2016, which focused on the detection of ESBL-producing Enterobacterales among healthy adult volunteers of a health science college, found a relatively low rate of 9.8% for fecal carriage of ESBL-producing *E. coli* and *Klebsiella* species [5]. A meta-analysis of data on ESBL carriage among healthy individuals found there was an increase in ESBL colonization of 5.38% per year [18] and this may have contributed to the high level of ESBL carriage found in the current study compared. Another reason for the higher prevalence of ESBL-producing isolates among HS students in the current study could have been due to the frequent visit of these student groups to healthcare settings which resulted in the acquisition of such bacteria from different hospital environments, patients, and healthcare workers.

In the current study, the majority of ESBL-producing Enterobacterales were *E. coli* (32.5%) with a higher rate of ESBL-producing *E. coli* isolated from HS students (46.5%, 47/101). All the ESBL-producing *Klebsiella* isolates were recovered from HS students (17.6%, 3/17). A variable rate of fecal carriage of ESBL-producing *E. coli* and *Klebsiella* species has been reported in community settings from different countries. The rate of ESBL production in *E. coli* was higher from a previous study in Nepal (77.8%) [6] and from one study from Egypt (62.7%) [19]. However, lower rates of ESBL carriage also have been reported from Nepal (7.87%) [5], France (6%) [20], UK (5.95%) [4], and Tunisia (7.3%) [21]. Previous reports have found 14.2% and 20.5% ESBL-producing *Klebsiella* species from the gut of Nepalese healthy population and student volunteers, respectively [5, 6]. These differences are likely to have been produced by different rates of exposure to antibiotics or to the bacterial isolates in the different populations. The students from the BNS program harbored a greater number of ESBL-producing *E. coli* (56.0%) and *Klebsiella* species (33.3%) than other groups of HS and NHS students, and this may also be attributed to a more frequent exposure of BNS students to ESBL-producing bacteria in hospitals before their enrollment in the BNS program as they would have undertaken job experience in hospitals prior to their enrollment in BNS program.

The current study also focused on the sensitivity of all *E. coli* and *Klebsiella* species isolates to a set of antimicrobial agents. When compared with non-ESBL producers, the ESBL-producers showed a higher resistance rate to ampicillin, cefotaxime, ceftazidime, ciprofloxacin, cotrimoxazole, and amikacin. This higher rates of resistance of ESBL-producers to quinolones, cotrimoxazole, and aminoglycosides is in accordance with other studies [15, 22]. The appearance of combined resistance to ampicillin and cotrimoxazole has become a common finding in ESBL-producing isolates in recent years as the genes responsible for resistance to these antibiotics are situated on the same plasmid [23]. None of the isolates in this study were found to be resistant to carbapenem. Mandal et al. [6] have also reported 100% sensitivity of ESBL-producing enterobacterial isolates from healthy populations towards imipenem and 4.85% ESBL-producing *E. coli* resistant to piperacillin-tazobactam while 25.0% and 75% *Klebsiella pneumoniae* resistant to ciprofloxacin and cotrimoxazole, respectively. Baral et al. [24] documented resistance of 60% and 11% against ceftazidime and amikacin, respectively, in ESBL-producing *E. coli* isolates from school children.

Fecal commensal Enterobacterales carrying ESBLs are becoming increasingly commonly isolated from the fecal microbiota of healthy adults. In developing countries such as Nepal, antibiotics are easily accessible and many people use antibiotics without medical advice. This may encourage spreading of antibiotic-resistant enterobacterial commensals. The frequent presence of resistant commensal isolates in the gut may be a risk factor for prolonged infections and also enable propagation of resistance to other bacteria [25].

## 5. Conclusions

An alarmingly high prevalence of intestinal carriage of ESBL-producing enterobacterial commensal isolates was detected among HS students. There was a considerable number of such bacteria colonizing NHS students as well. The results confirm that healthy individuals may be an important reservoir for ESBL-producing bacteria, and this may pose a risk for the transmission of resistance throughout the wider community. Therefore, there is a need to plan and implement policies that may reduce their prevalence. Similar types of studies should be conducted among different medical and health sciences students in different parts of the country and different countries to establish the extent of carriage of ESBL-producing bacteria among such populations.

### 5.1. Limitations

This study represents data from students of only two educational institutions in Nepal. Although this was a cross-sectional study among different student groups, we were unable to access the risk factors associated with this high rate of fecal carriage of ESBL-producing isolates. Molecular analysis of ESBL-producers could not be performed.

## Figures and Tables

**Table 1:** Distribution of *E. coli* and *Klebsiella* species among HS and NHS students.

Bacterial isolates	Number of isolates (%)		
	HS students	NHS students	Total
*Escherichia coli*	101 (49.8)	102 (50.2)	203
*Klebsiella pneumoniae*	8 (40.0)	12 (60.0)	20
*Klebsiella oxytoca*	9 (81.8)	2 (18.2)	11

Total	118 (50.4)	116 (49.6)	234

**Table 2:** Distribution of *E. coli* and *Klebsiella* species among different programs of HS students.

Bacterial isolates	Number of isolates (%)			
	B.Sc. Nursing	BNS	B.Sc. MLT	Total
*Escherichia coli*	57 (56.4)	25 (24.8)	19 (18.8)	101 (85.6)
*Klebsiella pneumoniae*	3 (37.5)	3 (37.5)	2 (25.0)	8 (6.8)
*Klebsiella oxytoca*	8 (88.9)	0 (0)	1 (11.1)	9 (7.6)

Total	68 (57.6)	28 (23.7)	22 (18.7)	118 (100)

**Table 3:** Prevalence of fecal carriage of ESBL-producing isolates.

Bacterial isolates	Number of isolates		Number of ESBL producers (%)			*p* value
	HS students	NHS students	HS students	NHS students	Total	
*E. coli* (*n* = 203)	101	102	47 (46.5)	19 (18.6)	66 (32.5)	<0.001
*Klebsiella* species (*n* = 31)	17	14	3 (17.6)	0 (0)	3 (9.7)	0.10

Total (*N* = 234)	118	116	50 (42.4)	19 (16.4)	69 (29.5)	<0.001

**Table 4:** ESBL carriage among different programs of HS students.

Bacterial isolates	Number of ESBL producers out of total number of isolates (%)			
	B.Sc. Nursing	BNS	B.Sc. MLT	Total
*E. coli*	26/57 (45.6)	14/25 (56.0)	7/19 (36.8)	47/101 (46.5)
*Klebsiella* species	2/11 (18.2)	1/3 (33.3)	0/3 (0)	3/17 (12.5)

Total (*n* = 118)	28/68 (41.2)	15/28 (22.1)	7/22 (31.8)	50/118 (42.4)

**Table 5:** Antibiotic resistance rates of bacterial isolates among HS and NHS students.

Antibiotics	*Escherichia coli*		*Klebsiella* species	
	HS students (*n* = 101)	NHS students (*n* = 102)	HS students (*n* = 17)	NHS students (*n* = 14)
Ampicillin	72 (71.3%)	52 (51.0%)	NA	NA
Piperacillin-tazobactam	2 (2.0%)	1 (1.0%)	1 (5.9%)	0 (0)
Cefotaxime	61 (60.4%)	31 (30.4%)	4 (23.5%)	0 (0)
Ceftazidime	61 (60.4%)	31 (30.4%)	4 (23.5%)	0 (0)
Ciprofloxacin	5 (5.0%)	7 (6.9%)	1 (5.9%)	0 (0)
Cotrimoxazole	40 (39.6%)	22 (21.6%)	5 (29.4%)	1 (7.1%)
Amikacin	4 (4.0%)	9 (8.8%)	1 (5.9%)	0 (0)
Meropenem	0 (0)	0 (0)	0 (0)	0 (0)

NA, not applicable.

**Table 6:** Antimicrobial resistance rates of ESBL-producing and non-producing isolates.

Antibiotics	ESBL-non-producers (*n* = 165)	ESBL producers (*n* = 69)	*p* value
Piperacillin-tazobactam	2 (1.2%)	2 (2.9%)	0.08
Ciprofloxacin	3 (1.8%)	10 (14.5%)	<0.001
Cotrimoxazole	27 (16.4%)	41 (59.4%)	<0.001
Amikacin	7 (4.2%)	7 (10.1%)	<0.001
Meropenem	0 (0)	0 (0)	

## Data Availability

Data will be made available if requested on justifiable reasons.

## Abbreviations

ASM: American Society for Microbiology
AST: Antibiotic susceptibility test
ATCC: American Type Culture Collection
BNS: Bachelor of Nursing Studies
B.Sc. MLT: Bachelor of Science in Medical Laboratory Technology
B.Sc. Nursing: Bachelor of Science in Nursing
CDT: Combined disk test
CLSI: Clinical and Laboratory Standards Institute
ESBL: Extended-spectrum *β*-lactamase
HS: Health science
MHA: Mueller-Hinton agar
NHS: Non-health science
ZOI: Zone of inhibition.
